# Acute effect of fine particulate matter and respiratory mortality in Changsha, China: a time-series analysis

**DOI:** 10.1186/s12890-022-02216-3

**Published:** 2022-11-12

**Authors:** Qi Feng, Yan Chen, Sha Su, Xixing Zhang, Xijian Lin

**Affiliations:** Changsha Center for Disease Control and Prevention, No. 509, Wanjiali Second North Road, Kaifu District, Changsha City, 410001 Hunan China

**Keywords:** Environmental exposure, PM_2.5_, Respiratory disease mortality, Time series, Epidemiology

## Abstract

**Background:**

Previous studies have confirmed that exposure to fine particulate matter (PM_2.5_) is associated with respiratory disease mortality. However, due to the differences in PM_2.5_ concentration, composition and population susceptibility within different regions, the estimates of the association between PM_2.5_ concentration and mortality are different. Moreover, few studies have examined the potential hazard of excessive PM_2.5_ exposure in terms of respiratory disease mortality.

**Methods:**

Daily recorded data on meteorological indices, environmental pollutants, and causes of death data in Changsha from January 2015 to December 2018 were obtained. The potential relationship between PM_2.5_ concentrations and respiratory disease mortality was determined using distributed lag nonlinear model (DLNM), which includes the relative risk (RR) and cumulative relative risk (CRR) of the lagged effect. The synergistic effects of other air pollutants were also considered.

**Results:**

A total of 8,825 cases of respiratory disease mortality occurred in Changsha between 2015 and 2018. The acute effect of PM_2.5_ concentration was associated with an increased risk of respiratory disease mortality. Regarding the lag specific effect, a 10 μg/m^3^ increase in PM_2.5_ concentration on respiratory disease mortality was statistically significant at lag day 0 and lag day 7 with a relative risk of 1.019 (95% CI 1.007- 1.031) and 1.013(95%CI: 1.002-1.024). As for the cumulative lag effect, a 4-day moving average of PM_2.5_ concentrations was significantly associated with a cumulative relative risk of 1.027 (95%CI: 1.011-1.031). The single-day lag effect and cumulative 4-day lag effect for male individuals were more significant than those observed in females. The effect of PM_2.5_ concentrations and respiratory disease mortality remained statistically significant in the multi-pollutant models (SO_2_, NO_2_, and O_3_). A higher risk was observed in the cold season than in the warm season.

**Conclusions:**

Our findings show a potential association between exposure to PM_2.5_ concentration and respiratory disease mortality in Changsha, with male individuals observed to have particularly higher risk.

## Introduction

Air pollution is a significant environmental and public health problem, particularly in developing countries. Throughout this past decade, numerous studies have provided substantial evidence for the relationship between air pollution and mortality [1–4]. Fine particulate matter (PM_2.5_, particulate matter ≤ 2.5 μm in aerodynamic diameter) is a major particulate matter pollutant, previous studies have confirmed that the deterioration of air quality is related to high concentrations of PM_2.5_, resulting in tremendous pressure on public health. The Global Burden of Disease study indicated that global deaths due to acute exposure to PM_2.5_ was 4.2 million (95% uncertainty interval 3.7 million to 4.8million) in 2015, compared with 3.5 million (95% uncertainty interval 3.0 million to 4.0 million) in 1990 [5]. Furthermore, adult cancer mortality data from 978 county-level surveillance sites in 31 Chinese provinces in 2014 showed that PM_2.5_ has become one of the 23 carcinogenic risk factors in China [6].

Compared with other organs, high concentrations of pollutants in the lungs and respiratory tract can cause considerable damage to the respiratory system. Moreover, the risk of death from respiratory diseases caused by PM_2.5_ is greater than that occurring due to non-accidental and cardiovascular mortality [7, 8]. In addition, earlier studies showed that short term exposure to PM_2.5_ exhibits a linear relationship with respiratory diseases mortality that differs from the relationship with non-accidental mortality and cardiovascular mortality [3, 9]. Therefore, it is necessary to study PM_2.5_ concentrations and respiratory disease mortality separately.

As the capital of China’s equipment manufacturing, Changsha is the central city of the Yangtze River Economic Belt and the political, economic, and cultural center of the Hunan Province. In recent years, owing to the efficacious economic development and the rise in energy consumption, air pollution has become an increasingly serious problem. Currently, most research focuses on individual large cities [10–12]. Due to differences in PM_2.5_ concentration, composition and population susceptibility within different regions, different countries or regions yield different estimates of the association between PM_2.5_ concentration and mortality [2, 13]. Therefore, relevant research in specific cities or regions is necessary.

In the present study, we collected data on meteorological, environmental pollutants and cause of death from January 2015 to December 2018 in Changsha. The purpose of this study is to assess the potential association between exposure to PM_2.5_ concentration and respiratory disease mortality in Changsha.

## Materials and methods

### Data collection

We collected daily respiratory disease mortality from the National population death information registration management system for residents in Changsha from January 1, 2015, to December 31, 2018. The cause of respiratory disease mortality was classified according to the International Classification of Disease, Revision 10 (ICD-10, J00–J99).

**Fig. 1:**
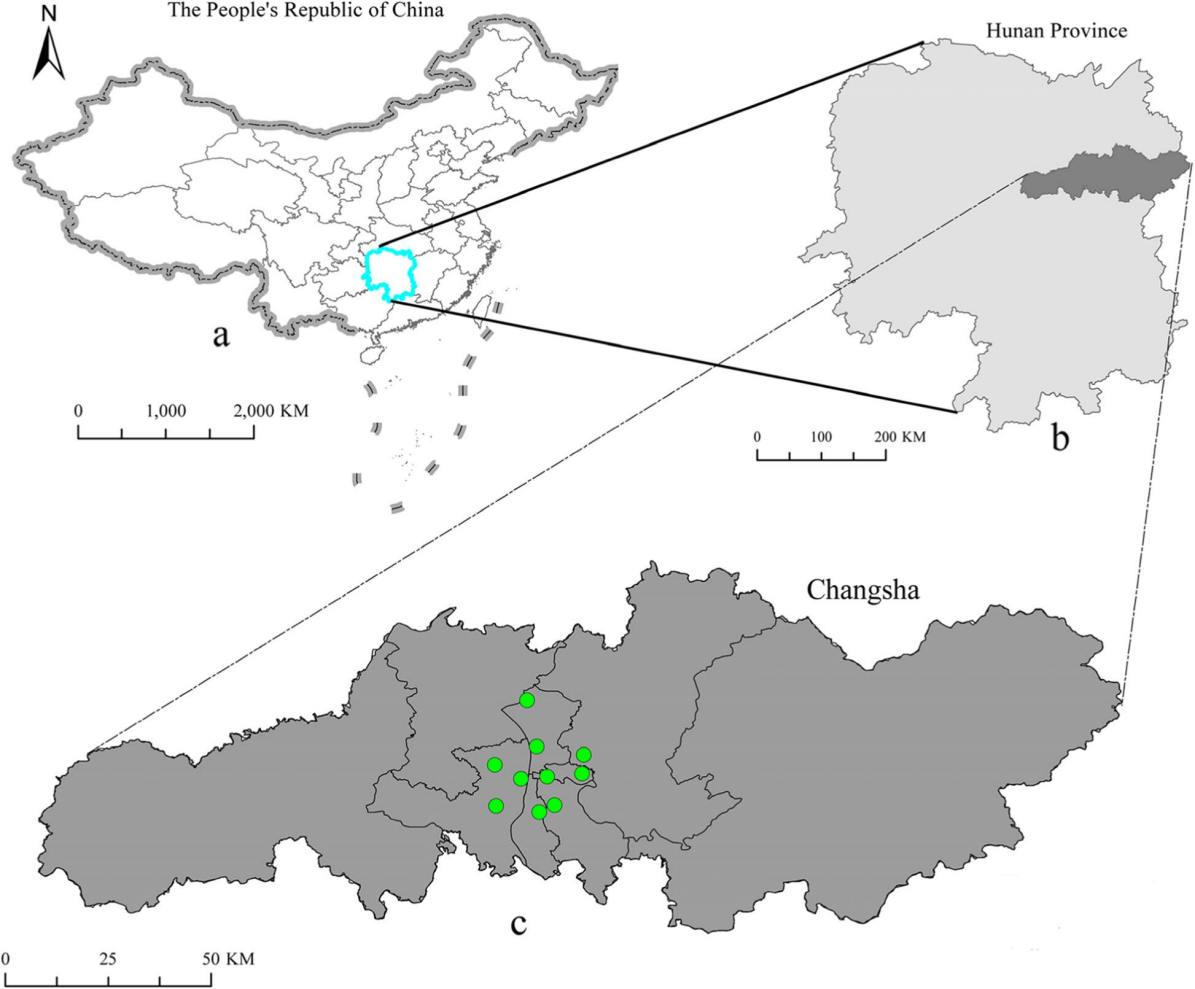
**a** Geographical location of Hunan Province in China. **b** Geographical location of Changsha city in Hunan Province. **c** Spatial distribution of air pollution monitoring stations in Changsha city. The green dots represent monitoring stations

We obtained PM_2.5_, inhalable particulate matter (PM_10_), sulfur dioxide (SO_2_), nitrogen dioxide (NO_2_), ozone (O_3_) data during the same period, which was collected and organized by the Changsha Environmental Protection Bureau. The air pollution data collected is the average of data from 10 environmental monitoring stations in the Changsha (Fig. 1). Average daily data collection requirements: ozone is the 8-h average concentration average, and other pollutants are the 24-h average concentration average. In addition, the environmental data in this article are publicly available on the website of the Ministry of Environment and Ecology of China (http://english.mee.gov.cn/).

We also collected daily temperature and relative humidity data of Changsha during the same period. The data comes from the Changsha Meteorological Bureau. The collected data are daily averages of all stations and can represent the city's average level. In addition, the meteorological data is publicly available on the website of the China Meteorological Administration (http://www.cma.gov.cn/en2014/).

### Statistical analysis

We used the mean ± standard deviation as maximum and minimum indicators to describe whole year or seasonal health data, and environmental data. We used raw scatter plots showing outcome (deaths) and exposure (PM_2.5_) data over time. The correlations between air pollutants and meteorological variables were analyzed using Spearman rank correlation.

R 3.6.2 software was used for the distributed lag nonlinear model [14]. A "cross-basis"(i.e., the tension product of the two functions of the exposure response relationship and the lag response relationship) was performed to establish a bi-dimensional space of a function, simultaneously describing the distribution of the dependent variable in the lag time dimension and the independent variable dimension. This process is performed in three steps, as follows: 1) establishing a basis cross matrix, 2) establishing a model based on the basis cross matrix, and 3) model prediction. The general model formula is as follows:

$${LogE(Y}_{t})={\sum }_{j=1}^{J}{{\beta }_{j}pollution}_{t-j}+ns\left({meteorological factor}_{t}, df=3\right)+ns\left(time, df=7*4\right)+{Dow}_{t}+{Holiday}_{t}+intercept$$

where *E (Y*_*t*_*)* is the outcome variable, which represents the expected number of deaths on day t. *β* represents the regression coefficients, pollution is described by PM_2.5_ and other air pollutants, ns represents the natural cubic splines, Dow is day of the week effect, and meteorological factor refer to relative humidity. Simultaneously, considering the lag effect of temperature on respiratory disease mortality, we established a cross-basis function of temperature as a control variable.Since the proposed model follows the quasi-Poisson distribution, and the degrees of freedom (df) follows the Akaike Information Criterion for Quasi-likelihood models (Quasi-AIC), we set different df values and performed sensitivity analysis by calculating the Quasi-AIC values of different models. The smaller the Quasi-AIC value, the better the model. The calculation formula of QAIC was as follows:

$$QAIC=-2ln(L)/c+2k$$

where L is the likelihood function, c is the variance expansion factor, and k is the number of model parameters. According to the Quasi-AIC principle, we adjusted the degrees of freedom for multiple model fittings; we used 7 df per year for the time variable, and 3 df for the relative humidity. The day of the week (DOW) and holidays(11 days per year) were included as dummy variables.

The health effects of air pollutants are sometimes not immediately observable and the effects may be delayed or even persist for a time. Therefore, we used different lag structures to estimate the lag specific effect and cumulative lag effect, including single-day (lag0 to lag7) and several-day moving averages (lag0-1 to lag0-7).

**Fig. 2:**
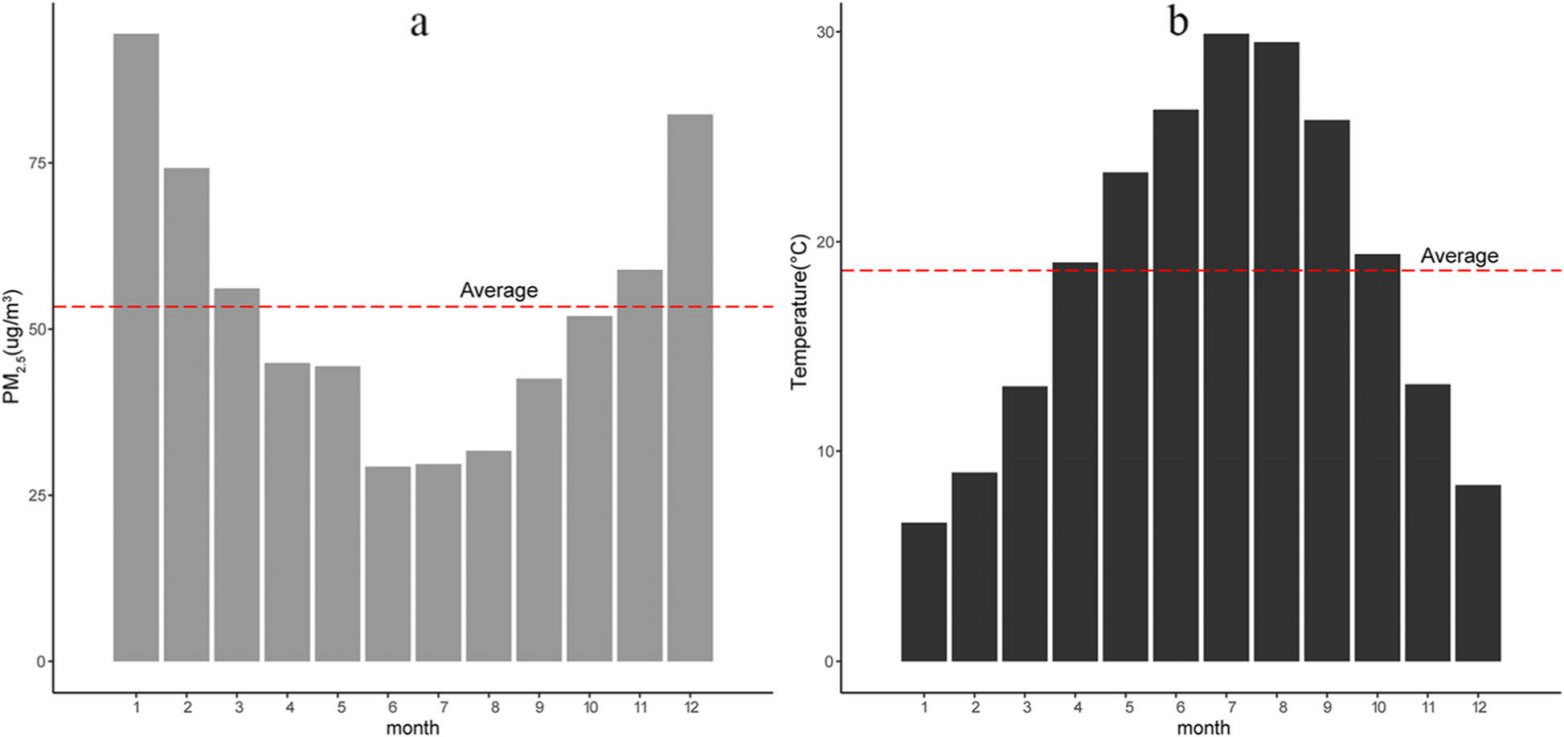
Monthly average PM_2.5_ concentration (**a**) and temperature bar graph (**b**) in Changsha city from January 1, 2015 to December 31, 2018. The horizontal red dotted line shows annual average concentration of PM_2.5_ (**a**) and temperature (**b**)

We first established a single-pollutant model of the relationship between daily PM_2.5_ concentrations and respiratory disease mortality risk. In addition to PM_2.5_ concentration, we focused on the other four indicators of air pollution: PM_10_, SO_2_, NO_2_, and O_3_, which were selected to identify typical air pollution concentrations. Air pollution mixtures have been considered in many cases because of their chemical and biological associations. Considering that the high collinearity between pollutants may lead to instability of the model, the correlation coefficient between PM_2.5_ concentration and other pollutants is less than 0.7 as the standard for introducing multi-pollutants. The warm (April–October) and cold (November-March) seasons were divided according to the annual average temperature (Fig. 2). Coincidentally, the average monthly concentration of PM_2.5_ in the warm season is below the annual average PM_2.5_ concentration, in the cold season, the reverse is true. Correspondingly, in seasonal classification analysis, we adjusted the spline function of the time variable to 3 df /year. In this study, the test level was set to α = 0.05.

## Results

The data on atmospheric pollutants, meteorological factors, and death of residents from respiratory disease in Changsha from January 1, 2015 to December 31, 2018 are shown in Table 1. The daily average concentrations of PM_2.5_, PM_10_, SO_2_, NO_2_, and O3 were 53.3 μg/m^3^, 73.0 μg/m^3^,14.2 μg/m^3^, 36.2 μg/m^3^, and 92 μg/m^3^, respectively. The concentrations of PM_2.5_, SO_2_, and NO_2_ were much higher in the cold season than in the warm season, while this trend was reversed for O_3_. A total of 8,825 cases of respiratory disease with reported mortality were obsed in the same period. As with seasonal trends in pollutant concentrations, the daily mortality was higher in the cold season (6.6 ± 3.4) than in the warm season (5.6 ± 3.4).

**Table 1:** Summary statistics daily levels of resident’s respiratory disease mortality and environmental indicators collected in Changsha, 2015–2018

Variable	Year-round (*N* = 1461)		Warm-season(April- October) (*N* = 856)		Cold-season(November -March) (*N* = 605)	
	Mean ± SD	Range(min, max)	Mean ± SD	range(min, max)	Mean ± SD	range(min, max)
PM_2.5_(μg/m^3^)	53.3 ± 35.2	(3,286)	39.2 ± 20.9	(3,160)	73.3 ± 41.3	(8,286)
PM_10_(μg/m^3^)	73.0 ± 41.7	(4,333)	62.8 ± 34.1	(4,257)	87.4 ± 46.9	(6,333)
SO_2_(μg/m^3^)	14.2 ± 7.7	(4,71)	12.7 ± 5.1	(4,42)	16.2 ± 9.9	(4,71)
NO_2_(μg/m^3^)	36.2 ± 16.3	(10,109)	29.3 ± 11.6	(10,73)	45.9 ± 17.1	(14,109)
O_3_(μg/m^3^)	92 ± 48.9	(7,260)	114.3 ± 45.0	(13,260)	60.5 ± 34.9	(7,200)
Temperature(°C)	18.67 ± 8.8	(-2.7,34.6)	24.8 ± 5.2	(9.6,34.6)	10.1 ± 4.7	(-2.7,25.1)
Relative humidity (%)	74.9 ± 14.0	(27,100)	74.4 ± 12.3	(40,99)	75.5 ± 16.1	(27,100)
Respiratory disease mortality	6.0 ± 3.4	(0,43)	5.6 ± 3.4	(0,43)	6.6 ± 3.4	(0,35)

Note: Cause of respiratory disease mortality was coded according to the International Classification of Disease, Revision 10 (ICD-10, J00–J99) (WHO 2016)

**Fig. 3:**
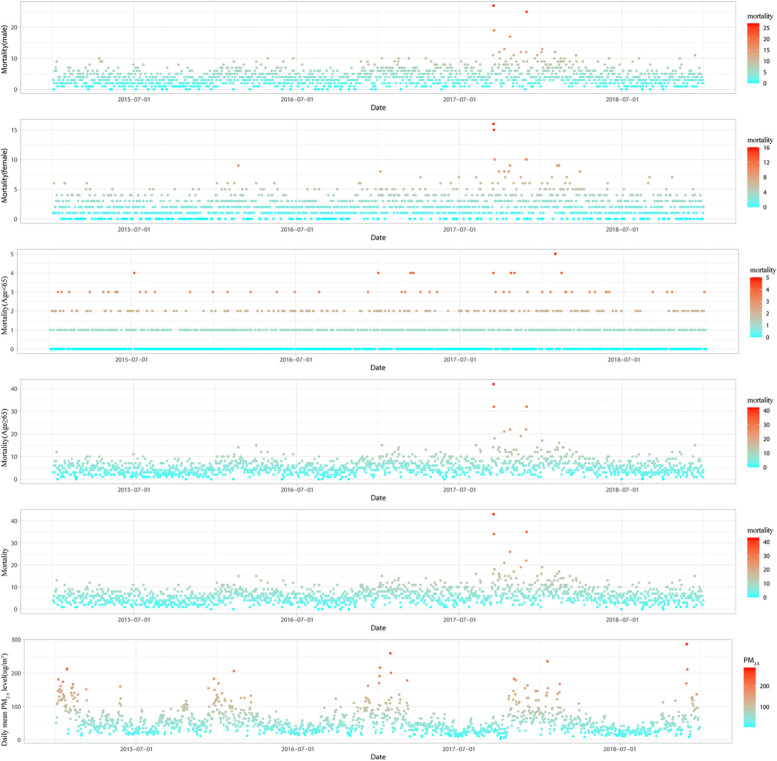
Raw plots showing outcome (mortality) and exposure (PM_2.5_) data over time

Figure 3 presents the raw plots for respiratory disease mortality and PM_2.5_ concentration, the subgroup of respiratory diseases mortality showed higher daily deaths among male individuals than among female individuals and higher mortality among individuals over 65 years of age than among those aged less than 65 years. Compared with the time series chart describing the number of deaths from respiratory diseases, the PM_2.5_ concentration showed a more obvious seasonal fluctuation, with the daily mean PM_2.5_ concentrations being much lower in the warm season than in the cold season.

Table 2 shows the Spearman correlations matrix between air pollutants and meteorological variables. PM_2.5_ concentration was positively correlated with PM_10_, SO_2_ and NO_2_, and negatively correlated with O_3_ concentration, air temperature and relative humidity. Additionally, the correlation between PM_2.5_ and each individual variable was statistically significant (*P* < 0.01).

**Table 2:** Spearman correlations between air pollutants and meteorological factors in Changsha, 2015–2018

Variables	PM_2.5_	PM_10_	SO_2_	NO_2_	O_3_	Temperature	Relative humidity
PM_2.5_	1.000	0.871^**^	0.508^**^	0.692^**^	-0.085^**^	-0.429^**^	-0.223^**^
PM_10_		1.000	0.691^**^	0.696^**^	0.137^**^	-0.183^**^	-0.510^**^
SO_2_			1.000	0.569^**^	0.108^**^	-0.012	-0.604^**^
NO_2_				1.000	-0.131^**^	-0.447^**^	-0.307^**^
O_3_					1.000	0.631^**^	-0.535^**^
Temperature						1.000	-0.253^**^
Relative humidity							1.000

Note: ** *P* < 0.01

**Fig. 4:**
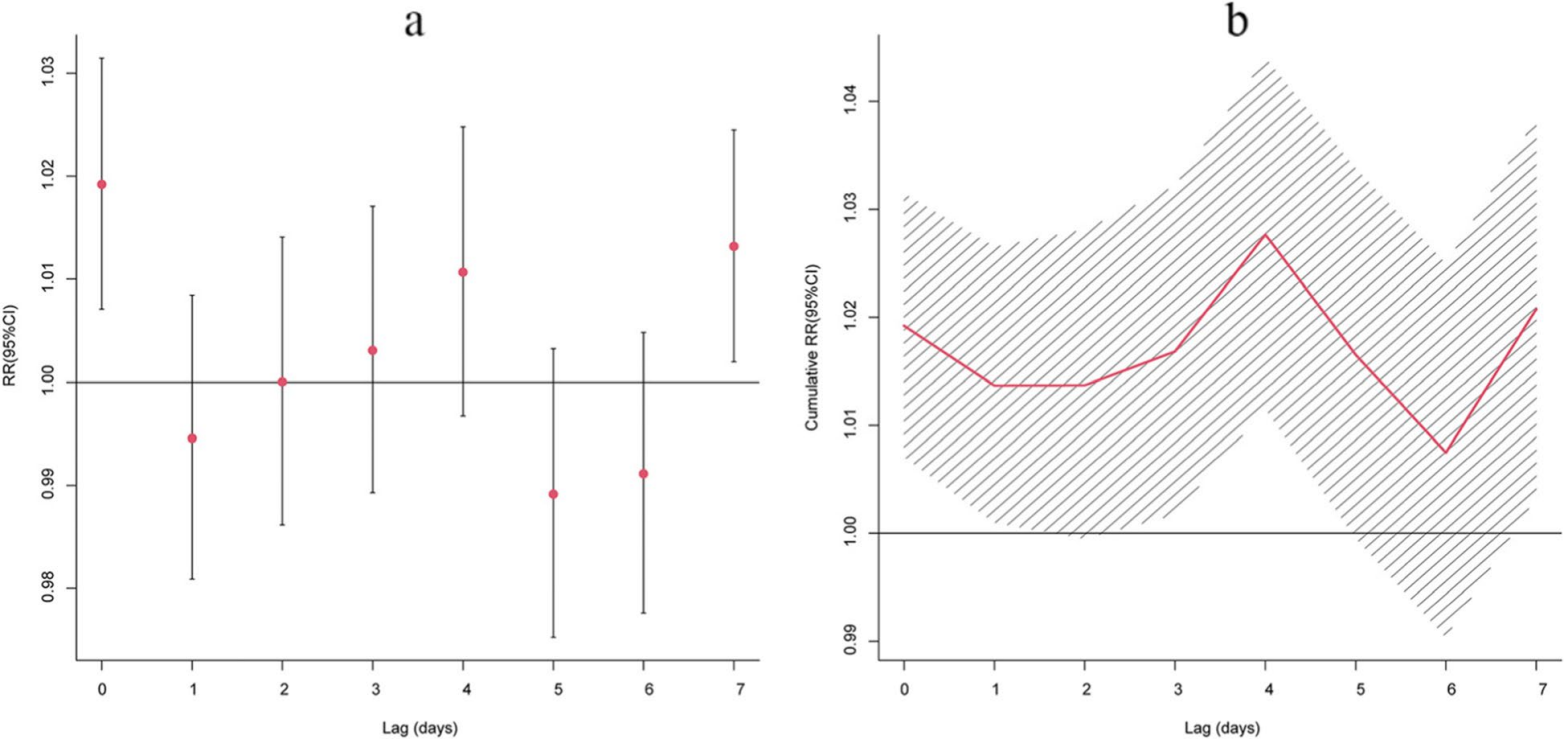
Lag-specific effects (**a**) and cumulative effects (**b**) of respiratory disease mortality for a 10 μg/m^3^ increase in PM_2.5_ in Changsha, China. 2015- 2018

We calculated single-day (lag 0–7) and cumulative (lag 01–07) lags for a 10 μg/m^3^ increase in PM_2.5_ concentration. After applying control for temperature, relative humidity, DOW, long-term trend of time (Fig. 4), we found that the impact of PM_2.5_ concentration on respiratory diseases mortality was statistically significant at lag day 0 and lag day 7, with a cumulative relative risk (lag0-4) of 1.027 (95%CI: 1.011-1.031).

Table 3 shows the daily respiratory disease mortality in every 10 μg/m^3^ increase of PM_2.5_ concentration for different sexes and age groups under different lag days. We observed that that the single-day lag effect and cumulative 4-day lag effect were both higher in male individuals than in female individuals. Moreover, the cumulative 4-day lag effect for those aged over 65 years was higher than that for those aged less than 65 years; on the contrary, the single-day lag effect was lower than that of those aged less than 65 years.

**Table 3:** The percent change of daily respiratory disease mortality in every 10 μg/m^3^ increase of PM_2.5_ to different genders and age groups under different lag days

Lag day	Total	Male	Female	Age < 65	Age ≥ 65
Lag0	1.019(1.007–1.031) ^*^	1.023(1.009–1.0367)^*^	1.013(0.994–1.0312)	1.027(0.997–1.057) ^*^	1.018(1.005–1.031) ^*^
Lag0-4	1.027(1.011–1.031) ^*^	1.032(1.014–1.0367) ^*^	1.018(0.994–1.0312)	1.018(1.005–1.031) ^*^	1.029(1.012–1.031) ^*^

Note: * *P* < 0.05

Table 4 shows the relative risks and cumulative relative risk of respiratory mortality associated with 10 μg/m^3^ increase in PM_2.5_ concentration under multiple pollutant models. The SO_2_, NO_2_, and O_3_ concentrations were substituted into the model for fitting to the multiple pollutant models and demonstrated similar lag curves in association with respiratory disease mortality. We observed that the lag-specific effect between PM_2.5_ concentration and respiratory mortality remained statistically significant and was not affected by other air pollutants. With regards to the cumulative effect, only the multiple pollutant models of PM_2.5_ and NO_2_ concentrations showed a relative increase.

**Table 4:** Relative risk and cumulative relative risk of respiratory disease mortality associated with 10 μg/m^3^ increase in PM_2.5_ concentration under different multi-pollutant model

Adjusting for pollutants	Lag0	95%CI	Lag0-4	95%CI
PM_2.5_				
+ SO_2_	1.018	(1.007–1.031)	1.024	(1.007–1.042)
+ NO_2_	1.019	(1.007–1.032)	1.032	(1.015–1.051)
+ O_3_	1.019	(1.006–1.031)	1.026	(1.009–1.044)
+ SO_2_ + NO_2_	1.019	(1.006–1.032)	1.029	(1.011–1.048)
+ SO_2_ + O_3_	1.018	(1.005–1.031)	1.023	(1.005–1.043)
+ NO_2_ + O_3_	1.019	(1.006–1.032)	1.031	(1.011–1.050)
+ SO_2_ + NO_2_ + O_3_	1.019	(1.006–1.032)	1.029	(1.010–1.049)

Note: The correlation coefficient between PM_2.5_ and PM_10_ is greater than 0.7 (0.871), considering the collinearity problem, we did not add PM_10_ to the multi-pollutant model for analysis

The analyses were adjusted for time trends, temperature, relative humidity, weekend, and holiday by using the distributed lag non-linear model

We simulated the relative risk and cumulative relative risk of respiratory disease mortality in association with different PM_2.5_ guidelines (Table 5). As the PM_2.5_ concentration increased, both the lag specific effect and cumulative lag effect increased significantly. When PM_2.5_ concentrations rose to 75 µg/m^3^, the relative risk of respiratory disease increased by 15.3% (1.153, 95%CI: 1.054–1.262), and the cumulative relative risk (lag0-4) increased by 22.7% (1.227, 95%CI: 1.090–1.262).

**Table 5:** Relative risk and cumulative relative risk of respiratory disease mortality from different PM_2.5_ guidelines

Guideline	Lag0	95%CI	Lag0-4	95%CI
WHO AQG2021	1.029	(1.011–1.048)	1.042	(1.017–1.048)
WHO AQG2005	1.049	(1.018–1.081)	1.071	(1.029–1.081)
GB Grade I	1.069	(1.025–1.115)	1.100	(1.041–1.115)
GB Grade II	1.153	(1.054–1.262)	1.227	(1.090–1.262)

Note: WHO AQG, The World Health Organization Air Quality Guideline 24-h daily average: 25 μg/m^3^(2005), 15 μg/m^3^(2021);

China National Ambient Air Quality Standards (2012)-Chinese 24-h standard, GB Grade I: 35 μg/m^3^, GB Grade II: 75 μg/m^3^

**Fig. 5:**
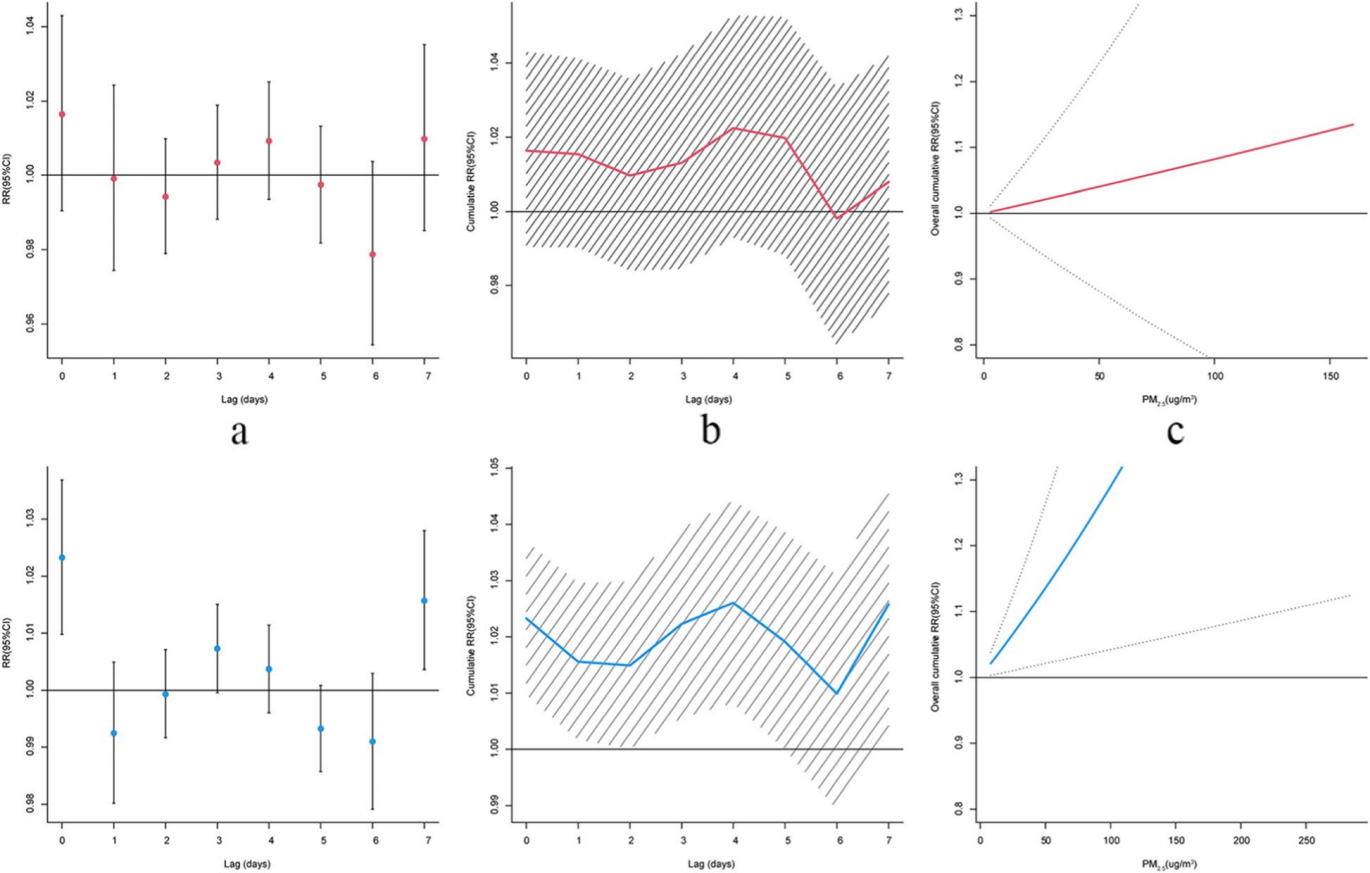
The effect of respiratory disease mortality for a 10 μg/m^3^ increase in PM_2.5_ in the warm season (top) and cold season (bottom). Lag-specific effects (**a**), cumulative effects (**b**) and overall cumulative association for 7 lags (**c**)

The association between PM_2.5_ concentration and respiratory mortality varies by season (Fig. 5). We observed stronger associations between PM_2.5_ concentration and respiratory disease mortality in the cold season, with a cumulative relative risk (lag0-4) of 1.026 (95%CI: 1.008–1.044). Although the lag effect was not statistically significant during the warm season, we found that the confidence interval for the overall cumulative effect was wider than that observed in the cold season.

## Discussion

Respiratory diseases are one of the leading causes of death in Chinese residents [15, 16]. In total, 8,825 case of respiratory disease mortality occurred in Changsha between 2015 and 2018. We calculated the lag specific and cumulative effects on respiratory mortality for each 10-μg/m^3^ increase in PM_2.5_ concentration. We found evidence of an association between PM_2.5_ concentration and respiratory disease mortality in Changsha. Furthermore, we observed clear evidence of associations between PM_2.5_ concentration, independent of other air pollutants, and respiratory disease mortality. We found that high concentrations of PM_2.5_ may pose a more serious risk to respiratory disease mortality. In addition, we also found PM_2.5_ concentration in the cold season was associated with a greater risk of respiratory disease mortality compared to that in the warm season.

According to environmental epidemiology, the effects of environmental exposure on health not only appear immediately but are also delayed [17, 18]. Therefore, we calculated the lag specific and cumulative lag effects comprehensively. In this study, we found significant associations between daily respiratory disease mortality and PM_2.5_ concentration at lag days 0 and 7, and a cumulative relative risk (lag0-4) of 1.027 (95%CI: 1.011-1.031). A study of 652 cities across the globe reported that a 10 μg/m^3^ increase in PM_2.5_ concentration was associated with a 0.74% increase (95%CI, 0.53–0.95) in respiratory disease mortality [1]. In a U.S. study of 75 cities, there was a 1.18% (95%CI, 0.93–1.44%) increase in all-cause mortality and a 1.71% (95%CI 1.06- 2.35%) increase in respiratory disease-related deaths [8]. Another multicity time-series study in East Asia found that each 10-μg/m^3^ increase in PM_2.5_ concentration (lag01) was associated with a 1% increase (95%CI 0.23 -1.78%) in respiratory disease mortality [19]. In Guangzhou, Wu et al. found that 2-day moving average (lag01) PM_2.5_ concentration corresponded to 0.95% (95% CI 0.16–1.73%) increase in respiratory disease mortality[12]. One study from Shanghai noted evidence of stronger associations between ambient particulate matter and respiratory disease mortality in individuals aged > 65 years [11]. Part of our results are inconsistent with those of previous studies. First, this may be related to the difference in PM_2.5_ concentration [20]; in contrast, Zhang et al. observed a stronger association between the level of particulate matter and the rate of mortality. Considering that China has experienced unprecedented severe air pollution events since 2011, with northern regions most affected, the discrepancies in our results might be, at least in part, due to the relatively southern position of Changsha [7]. Secondly, these inconsistencies may be due to the different toxic components of PM_2.5_. Several studies have found that coal combustion emissions may have a more significant toxic effect, particularly on the respiratory system [8, 13]. Yin et al. reported that northern China had generally higher coal combustion emissions than those found in southern China because of more intense industrial emissions by thermal power generation, for instance [21]. Finally, differences related to the ethnicity, age, physical constitution, and population tolerance to pollutants within different regions may contribute to the discrepancies observed in our study.

Previous studies have also considered age and sex to be important factors contributing to the impact of PM_2.5_ concentration on respiratory disease mortality. Likewise, we note significant sex differences influencing the effects of PM_2.5_ concentration on respiratory disease mortality in male individuals; each 10-μg/m^3^ increase in PM_2.5_ concentration increased the risk of respiratory disease mortality by 2.3% and cumulative risk of mortality by 3.2%. Results from large nationwide cohort of Chinese males [21] reported that each 10-μg/m^3^ increase in PM_2.5_ concentration was associated with a 9%, 12% and 12% increase in the risk of death due to non-accidental conditions, chronic obstructive pulmonary disease, and lung cancer, respectively. Zeng et al. suggested that younger populations are more vulnerable to exposure to PM_2.5_ [22]. One study found that in terms of age, PM_2.5_ exposure was significantly associated with respiratory disease mortality in the subgroup aged ≤ 64 years, while there was no significant risk in the subgroup aged ≥ 65 years [23]. However, some studies suggest that sensitive individuals aged older than 65 years have a higher risk of death, even at low PM_2.5_ concentrations [24]. The findings of the current study illustrate that individuals aged < 65 years had a higher respiratory diseases risk of death in a single day, and while the cumulative risk of death was higher, the risk of death was lower in individuals older than 65 years as compared to risk in younger individuals. Consistent with findings from other studies [7, 25], the described differences in age and sex are thought to be due to the diverse nature pertaining to the subgroups of disease types and environmental factors sensitivities [26].In addition, the present study reports that while the cumulative relative risk (lag0-4) is evidently higher for older individuals, the single-day lag effect is stronger for the younger ones. The probable cause is hereby ascribed to the coexistence of chronic diseases and multiple conditions in the elderly. Hence, the harmful consequences of PM_2.5_ to a single organ or system may also be involved implicated in multi-organ and multi-system diseases, resulting in persistent damage.

Air quality guidelines have been developed to guide policies on addressing efforts to reduce the health impact of air pollution in the population. Epidemiological studies have confirmed that when reaching a certain concentration, air pollution alters the corresponding morbidity and mortality of the respective population. The World Health Organization (WHO) Air Quality Guideline (AQG) estimates a critical value causing health hazards, and recommends that the atmospheric environmental quality standards in the guidelines be used as threshold. Most countries, including China, have not yet reached the WHO 2005 AQG [27] guidance value; furthermore, the latest WHO 2021 AQG [28] guidance value brings to light greater challenges to air pollution control globally. Chien et al. reported that a PM_2.5_ index greater than 35 μg/m^3^ affects sensitive populations, and the spline for PM_2.5_ concentration was centered at this threshold [17]. A 2019 study using concentration**–**response functions estimated excess deaths at three counterfactual PM_2.5_ thresholds (7.5, 25, and 75 µg/m^3^) [9]. Here, we compared the difference in mortality risk at four different PM_2.5_ guidelines: WHO AQG 2021(15 μg/m^3^), WHO AQG 2005(25 μg/m^3^), Grade I (35 µg/m^3^) and Grade II (75 µg/m^3^) national standards outlined by the National Ambient Air Quality Standards of China [29]. In addition to other health factors, we found that high PM_2.5_ concentrations may lead to a more serious risk of respiratory disease mortality in this study. A previous report concluded that when estimating health effects under a population-weighted PM_2.5_ threshold, setting more specific emission limits or penalties for controlling pollution activities in China and other developing countries can be considered [30]. It is worth noting that many countries have not adopted the WHO guidelines. The United States revised the PM_2.5_ standard in 2006 [31], reporting annual and daily average values were 15 μg/m^3^ and 35 μg/m^3^, respectively, which did not meet WHO guidelines. Given that standard setters must balance air pollution levels, health risks, economic capabilities, and comprehensive governance capabilities, air quality standards vary across different countries and regions. PM_2.5_ concentration was included in the National Ambient Air Quality Standards for the first time in 2012, although it did not meet the WHO standards [27] or those of other countries [31–33]. Nevertheless, this reflects progress of the Chinese government in protecting public health.

Consistent with previously reported data [34], we found PM_2.5_ concentration in the cold season to be associated with a higher risk of respiratory disease mortality than that noted in the warm season. Cohort studies have confirmed that higher PM_2.5_ concentrations result in a greater observed rates of respiratory disease mortality [21]. A review reported that pollutant emissions increased during the central heating period (November to March) in northern China [9]. Furthermore, some scholars believe that the PM_2.5_ concentration in the cold season corresponds to a higher risk of respiratory diseases mortality in consequence of its relationships with low temperature and atmospheric inversion [35, 36]. These effects are more pronounced in southern China [37].Therefore, we used meteorological factors, such as temperature and humidity to act as control variables in our study, and our results remained significant. In addition, the frequent occurrence of severe pollution during the cold season is related to the climate and topographical characteristics of Changsha. Changsha has a subtropical monsoon humid climate, with northwest wind dominating the cold season. The terrain altitude of Changsha is high in the south and low in the north, forming a horseshoe shaped opening to the north, causing air pollutants to remain trapped.

Although the acute health hazards of particulate pollutants in high concentration during the cold season are more significant, the long-term hazards of low concentration cannot be ignored [38, 39]. Both short-term and long-term exposure to particulate pollutants in low concentration ranges are linked with adverse health effects. We found that although the relative risk values were indeed lower during the warm season, the 95% confidence intervals overlapped considerably. Moreover, we found that the concentration of fine particulate matter in the warm season is far from the average concentration (53.3 μg/m^3^) and that the range of the confidence interval significantly widens. These findings highlight the impacts that short-term exposure and low concentrations of particulate pollutants have over respiratory disease mortality rates. According to twenty-two European cohorts observed in studies assessing air pollution effects, the relative hazard ratio of population exposure to PM_2.5_ remains high, even with long-term exposure below the European average annual limit (25 μg/m^3^) [40]. A U.S. study found statistically significant association between short-term exposures to PM_2.5_ and the relative hazard to the population, with a relative increase of 1.05% (95% CI, 0.95%-1.15%) during 2000–2012, even though the average daily concentration of PM_2.5_ was below 25 μg/m^3^ for 93.6% of the days recorded [24].However, nowadays more individuals wear masks, close windows, and reduce outdoor activities on heavily polluted days. Nonetheless, few studies have examined the health effects of low-level exposures and lax prevention.

Our study had several limitations. First, the research methods were limited, compared with extensive sample epidemiological cohort studies. Time-series studies use ecological methods. Their power and reliability are limited in inferring the causal relationship between air pollution and health. Second, the potential underreporting of death data from the cause of death monitoring system used may have had an impact on the results, whereas the fixed-point monitoring data may have introduced a bias in analyzing the exposure levels of the city's population. Third, the study did not include a questionnaire on factors, such as lifestyle, disease history, or other influencing factors that could not be studied or analyzed as control variables.

## Conclusion

Our study demonstrated an association between PM_2.5_ concentrations and respiratory disease mortality in Changsha, China. Younger people (age < 65 years) and males might be more vulnerable to exposure to PM_2.5_, while older people have a higher cumulative risk of death; therefore, the protective measures for specific groups should be strengthened. In addition, we found stronger associations between air pollutants and respiratory disease mortality during the cold season compared to those observed during the warm season. Regardless, the harm caused by low-concentration particulate matter during the warm season should not be ignored. In view of the high concentration of PM_2.5_ that may pose a more serious risk of respiratory disease mortality, we believe that relevant government departments should start a new round of standard revision as soon as possible under the dual goals of promoting carbon neutrality and continuously improving air quality.

## Data Availability

The environmental data (PM_2.5_, NO_2_, SO_2_ and O_3_) in this article are publicly available on the website of the Ministry of Environment and Ecology of China (http://english.mee.gov.cn/).Meteorological data (temperature, relative humidity) is publicly available on the website of the China Meteorological Administration (http://www.cma.gov.cn/en2014/). The death data that support the findings of this study are available from the CDC's population death information registration management system, but restrictions apply to the availability of these data, which were used under license for the current study. In addition, other ways to obtain the death data: 1. Contact the Center for Chronic and Non-Communicable Diseases of the Chinese Center for Disease Control and Prevention (e-mail: ncncdoffice@chinacdc.cn), 2. Purchase the annual "China Death Cause Surveillance Dataset" compiled by the Chinese Center for Disease Control and Prevention. 3. The China Public Health Science Data Center is regularly published (https://www.phsciencedata.cn/Share/en/index.jsp).

## Abbreviations

PM_2.5_: Fine particulate matter
RR: Relative risk
CRR: Cumulative relative risk
DLNM: Distributed lag nonlinear model
PM_10_: Inhalable particulate matter
SO_2_: Sulfur dioxide
NO_2_: Nitrogen dioxide
O_3_: Ozone
Quasi-AIC: Akaike Information Criterion for Quasi-likelihood models
DOW: Day of the week
AQG: Air Quality Guideline

## References

[1] Liu C, Chen R, Sera F, Vicedo-Cabrera AM, Guo Y, Tong S, Coelho M, Saldiva PHN, Lavigne E, Matus P (2019). Ambient Particulate Air Pollution and Daily Mortality in 652 Cities. N Engl J Med.

[2] Chen R, Yin P, Meng X, Wang L, Liu C, Niu Y, Liu Y, Liu J, Qi J, You J (2019). Associations between Coarse Particulate Matter Air Pollution and Cause-Specific Mortality: A Nationwide Analysis in 272 Chinese Cities. Environ Health Perspect.

[3] Burnett R, Chen H, Szyszkowicz M, Fann N, Hubbell B, Pope CA, Apte JS, Brauer M, Cohen A, Weichenthal S (2018). Global estimates of mortality associated with long-term exposure to outdoor fine particulate matter. Proc Natl Acad Sci U S A.

[4] Laden F, Schwartz J, Speizer FE, Dockery DW (2006). Reduction in fine particulate air pollution and mortality: Extended follow-up of the Harvard Six Cities study. Am J Respir Crit Care Med.

[5] Cohen AJ, Brauer M, Burnett R, Anderson HR, Frostad J, Estep K, Balakrishnan K, Brunekreef B, Dandona L, Dandona R (2017). Estimates and 25-year trends of the global burden of disease attributable to ambient air pollution: an analysis of data from the Global Burden of Diseases Study 2015. The Lancet.

[6] Chen W, Xia C, Zheng R, Zhou M, Lin C, Zeng H, Zhang S, Wang L, Yang Z, Sun K (2019). Disparities by province, age, and sex in site-specific cancer burden attributable to 23 potentially modifiable risk factors in China: a comparative risk assessment. Lancet Glob Health.

[7] Zhang J, Liu Y, Cui LL, Liu SQ, Yin XX, Li HC (2017). Ambient air pollution, smog episodes and mortality in Jinan. China Sci Rep.

[8] Dai L, Zanobetti A, Koutrakis P, Schwartz JD (2014). Associations of fine particulate matter species with mortality in the United States: a multicity time-series analysis. Environ Health Perspect.

[9] Yan M, Wilson A, Bell ML, Peng RD, Sun Q, Pu W, Yin X, Li T, Anderson GB (2019). The Shape of the Concentration-Response Association between Fine Particulate Matter Pollution and Human Mortality in Beijing, China, and Its Implications for Health Impact Assessment. Environ Health Perspect.

[10] Luo K, Li W, Zhang R, Li R, Xu Q, Cao Y (2016). Ambient Fine Particulate Matter Exposure and Risk of Cardiovascular Mortality: Adjustment of the Meteorological Factors. Int J Environ Res Public Health.

[11] Kan H, London SJ, Chen G, Zhang Y, Song G, Zhao N, Jiang L, Chen B (2007). Differentiating the effects of fine and coarse particles on daily mortality in Shanghai. China Environment International.

[12] Wu R, Zhong L, Huang X, Xu H, Liu S, Feng B, Wang T, Song X, Bai Y, Wu F (2018). Temporal variations in ambient particulate matter reduction associated short-term mortality risks in Guangzhou, China: A time-series analysis (2006–2016). Sci Total Environ.

[13] Achilleos S, Kioumourtzoglou MA, Wu CD, Schwartz JD, Koutrakis P, Papatheodorou SI (2017). Acute effects of fine particulate matter constituents on mortality: A systematic review and meta-regression analysis. Environ Int.

[14] Gasparrini A, Armstrong B, Kenward MG (2010). Distributed lag non-linear models. Stat Med.

[15] Ren Z, Liu X, Liu T, Chen D, Jiao K, Wang X, Suo J, Yang H, Liao J, Ma L (2021). Effect of ambient fine particulates (PM2.5) on hospital admissions for respiratory and cardiovascular diseases in Wuhan, China. Respir Res.

[16] Zhou M, Wang H, Zeng X, Yin P, Zhu J, Chen W, Li X, Wang L, Wang L, Liu Y (2019). Mortality, morbidity, and risk factors in China and its provinces, 1990–2017: a systematic analysis for the Global Burden of Disease Study 2017. The Lancet.

[17] Chien LC, Guo Y, Li X, Yu HL (2018). Considering spatial heterogeneity in the distributed lag non-linear model when analyzing spatiotemporal data. J Expo Sci Environ Epidemiol.

[18] Liu XB, Wen XM, Sun XH, Hong QQ, Wang Q, Kang Z, Xia SJ, Yang C, Zhu S (2021). The Short-Term Effects of Ambient Air Pollutants are Associated With Daily Mortality in Northeast China From 2014 to 2018: A Time Series Analysis. J Occup Environ Med.

[19] Lee H, Honda Y, Hashizume M, Guo YL, Wu CF, Kan H, Jung K, Lim YH, Yi S, Kim H (2015). Short-term exposure to fine and coarse particles and mortality: A multicity time-series study in East Asia. Environ Pollut.

[20] Ye R, Cui L, Peng X, Yu K, Cheng F, Zhu Y, Jia C (2019). Effect and threshold of PM2.5 on population mortality in a highly polluted area: a study on applicability of standards. Environ Sci Pollut Res Int.

[21] Yin P, Brauer M, Cohen A, Burnett RT, Liu J, Liu Y, Liang R, Wang W, Qi J, Wang L, Zhou M (2017). Long-term Fine Particulate Matter Exposure and Nonaccidental and Cause-specific Mortality in a Large National Cohort of Chinese Men. Environ Health Perspect.

[22] Zeng W, Zhang Y, Wang L, Wei Y, Lu R, Xia J, Chai B, Liang X (2018). Ambient fine particulate pollution and daily morbidity of stroke in Chengdu. China PLoS One.

[23] Mokoena KK, Ethan CJ, Yu Y, Shale K, Liu F (2019). Ambient air pollution and respiratory mortality in Xi'an, China: a time-series analysis. Respir Res.

[24] Di Q, Dai L, Wang Y, Zanobetti A, Choirat C, Schwartz JD, Dominici F (2017). Association of Short-term Exposure to Air Pollution With Mortality in Older Adults. JAMA.

[25] Rodríguez-Villamizar LA, Rojas-Roa NY, Blanco-Becerra LC, Herrera-Galindo VM, Fernández-Niño JA (2018). Short-Term Effects of Air Pollution on Respiratory and Circulatory Morbidity in Colombia 2011-2014: A Multi-City, Time-Series Analysis. Int J Environ Res Public Health.

[26] Lee SW, Yon DK, James CC, Lee S, Koh HY, Sheen YH, Oh JW, Han MY, Sugihara G (2019). Short-term effects of multiple outdoor environmental factors on risk of asthma exacerbations: Age-stratified time-series analysis. J Allergy Clin Immunol.

[27] WHO: WHO Air Quality Guidelines for Particulate Matter, Ozone, Nitrogen, Dioxide and Sulfur Dioxide. Global update 2005, Summary of Risk Assessment. 2005.

[28] WHO: WHO global air quality guidelines: particulate matter (PM2.5 and PM10), ozone, nitrogen dioxide, sulfur dioxide and carbon monoxide . 2021. 34662007

[29] Ministry of Ecological Environment of the People's Republic of China .National Ambient Air Quality Standards.2012. [http://www.mee.gov.cn/ywgz/fgbz/bz/bzwb/dqhjbh/dqhjzlbz/201203/t20120302_224165.shtml]

[30] Tran BL, Chang CC, Hsu CS, Chen CC, Tseng WC, Hsu SH (2019). Threshold Effects of PM2.5 Exposure on Particle-Related Mortality in China. Int J Environ Res Public Health.

[31] United States Environmental Protection Agency. Process of Reviewing the National Ambient Air Quality Standards. 2012. [https://www.epa.gov/criteria-air-pollutants/naaqs-table]

[32] Ministry of the Environment Government of Japan. Environmental Quality Standards in Japan - Air Quality. 2009. [http://www.env.go.jp/en/air/aq/aq.html]

[33] The Ministry of Environment, Forest and Climate Change .National ambient air quality standards. 2009. [http://www.indiaenvironmentportal.org.in/content/291467/revised-national-ambient-air-quality-standards-naaqs-2009/]

[34] Hvidtfeldt UA, Sorensen M, Geels C, Ketzel M, Khan J, Tjonneland A, Overvad K, Brandt J, Raaschou-Nielsen O (2019). Long-term residential exposure to PM2.5, PM10, black carbon, NO2, and ozone and mortality in a Danish cohort. Environ Int.

[35] Li Y, Zheng C, Ma Z, Quan W (2019). Acute and Cumulative Effects of Haze Fine Particles on Mortality and the Seasonal Characteristics in Beijing, China, 2005–2013: A Time-Stratified Case-Crossover Study. Int J Environ Res Public Health.

[36] Dang TN, Seposo XT, Duc NH, Thang TB, An DD, Hang LT, Long TT, Loan BT, Honda Y (2016). Characterizing the relationship between temperature and mortality in tropical and subtropical cities: a distributed lag non-linear model analysis in Hue, Viet Nam, 2009–2013. Glob Health Action.

[37] Zhang Y, Wang S, Zhang X, Ni C, Zhang J, Zheng C (2020). Temperature modulation of the adverse consequences on human mortality due to exposure to fine particulates: A study of multiple cities in China. Environ Res.

[38] Atkinson RW, Kang S, Anderson HR, Mills IC, Walton HA (2014). Epidemiological time series studies of PM2.5 and daily mortality and hospital admissions: a systematic review and meta-analysis. Thorax.

[39] Schwartz J, Bind MA, Koutrakis P (2017). Estimating Causal Effects of Local Air Pollution on Daily Deaths: Effect of Low Levels. Environ Health Perspect.

[40] Beelen R, Raaschou-Nielsen O, Stafoggia M, Andersen ZJ, Weinmayr G, Hoffmann B, Wolf K, Samoli E, Fischer P, Nieuwenhuijsen M (2014). Effects of long-term exposure to air pollution on natural-cause mortality: an analysis of 22 European cohorts within the multicentre ESCAPE project. The Lancet.

